# A case report: cccDNA and pgRNA remain positive in liver tissue in a chronic hepatitis B patient with functional cure

**DOI:** 10.3389/fmed.2024.1427043

**Published:** 2024-10-29

**Authors:** Wenxiong Xu, Qiumin Luo, Yeqiong Zhang, Chan Xie, Liang Peng

**Affiliations:** ^1^Department of Infectious Diseases, The Third Affiliated Hospital of Sun Yat-sen University, Guangzhou, China; ^2^Guangdong Key Laboratory of Liver Disease Research, The Third Affiliated Hospital of Sun Yat-sen University, Guangzhou, China; ^3^Key Laboratory of Tropical Disease Control, Ministry of Education, Sun Yat-sen University, Guangzhou, China

**Keywords:** hepatitis B surface antigen, covalently closed circular deoxyribonucleic acid, pregenomic ribonucleic acid, functional cure, chronic hepatitis B

## Abstract

Hepatitis B surface antigen (HBsAg) seroclearance is recommended as the ideal endpoint for nucleos(t)ide analog (NA) treatments. Functional cure of chronic hepatitis B (CHB) is defined as having undetectable serum hepatitis B virus (HBV) deoxyribonucleic acid and serum HBsAg. We report a functional cure case of CHB with a family history of hepatocellular carcinoma (HCC) after long-term NA therapy. Despite achieving functional cure for over 7 years, both HBV covalently closed circular deoxyribonucleic acid (cccDNA) and pregenomic ribonucleic acid (pgRNA) remain positive in the liver tissue of the patient, indicating that a sterilizing cure has not been achieved. This case highlights the importance of active surveillance of HBV cccDNA and pgRNA for sterilizing the cure and risk of HCC.

## Introduction

Globally, two billion people are infected by the hepatitis B virus (HBV) (1). Antiviral drugs for chronic hepatitis B (CHB) currently include pegylated interferon-*α* (Peg-IFN-α) and nucleos(t)ide analogs (NAs) (2). NA treatment can efficiently reduce blood HBV deoxyribonucleic acid (DNA) levels (3). However, hepatitis B surface antigen (HBsAg), produced by the translation of messenger ribonucleic acid (mRNA) generated from transcriptionally active covalently closed circular DNA (cccDNA) and integrated HBV DNA sequences (4), is often difficult to eliminate with NA monotherapy (5). HBsAg seroclearance is recommended as the ideal endpoint for NA treatments by guidelines for the prevention and treatment of CHB of the Chinese Medical Association (6), the Asian Pacific Association for the Study of the Liver (2), the European Association for the Study of the Liver (7), the American Association for the Study of Liver Diseases (8), and the World Health Organization (9). A functional cure is defined as having undetectable serum HBV DNA and serum HBsAg, with or without HBsAg seroconversion, regardless of cccDNA silencing (10). This differs from a sterilizing cure, in which both cccDNA and HBsAg are eliminated from hepatocytes. We report a functional cure case of CHB with hepatocellular carcinoma (HCC) family history after long-term NA therapy. Both cccDNA and pregenomic ribonucleic acid (pgRNA) remain positive in liver tissue, despite a functional cure that has been achieved for over 7 years.

## Case presentation

A female patient was diagnosed with HBV infection by a routine health examination 20 years ago. An examination of blood HBV biomarkers indicated positive HBsAg, hepatitis B e antibody, hepatitis B core antibody, and HBV DNA, while hepatitis B surface antibody (HBsAb) and hepatitis B e antigen were negative. Since she had no discomfort and the alanine aminotransferase (ALT) level was in the normal range, regular tests, such as liver enzymes, HBV biomarkers, and liver ultrasound, were performed thereafter without anti-HBV treatment. She disclosed that she had a family history of HBV infection and HCC. Her father and aunt were chronically infected by HBV. Her aunt was diagnosed with HCC.

On 27 February 2013, an occasional abdomen ultrasonic examination for the patient showed scattered patchy abnormal hyper-echoic masses in the liver and slight splenomegaly, indicating the possibility of liver cirrhosis. A liver biopsy targeting the masses was performed on 11 March 2013. Some hepatocytes showed hydropic degeneration. Spotty necrosis and focal necrosis could be easily seen. Some areas showed pseudolobule formation trends. The results showed chronic hepatitis with inflammation staging “G2” and fibrosis staging “S3.” Both the blood cell count and ALT levels were in the normal range, and the level of HBV DNA was 37,900 IU/mL. Alpha-fetoprotein (AFP) was negative. Anti-HBV treatment of oral telbivudine 600 mg per day was initiated at the Department of Infectious Diseases, the Third Affiliated Hospital of Sun Yat-sen University. Negative HBV DNA was achieved on 26 July 2013. HBsAg clearance was achieved on 16 June 2016. Positive HBsAb was achieved on 8 March 2018. AFP remained negative in the course. Regular abdomen ultrasonic examinations showed similar results to those obtained on 27 February 2013. As the patient achieved a functional cure and HBsAg seroconversion, telbivudine was discontinued on 8 March 2018. ALT normalization, HBV DNA clearance, HBsAg seroconversion, and AFP negativity were maintained in the 5-year follow-up (from March 2018 to August 2023).

**Figure 1:**
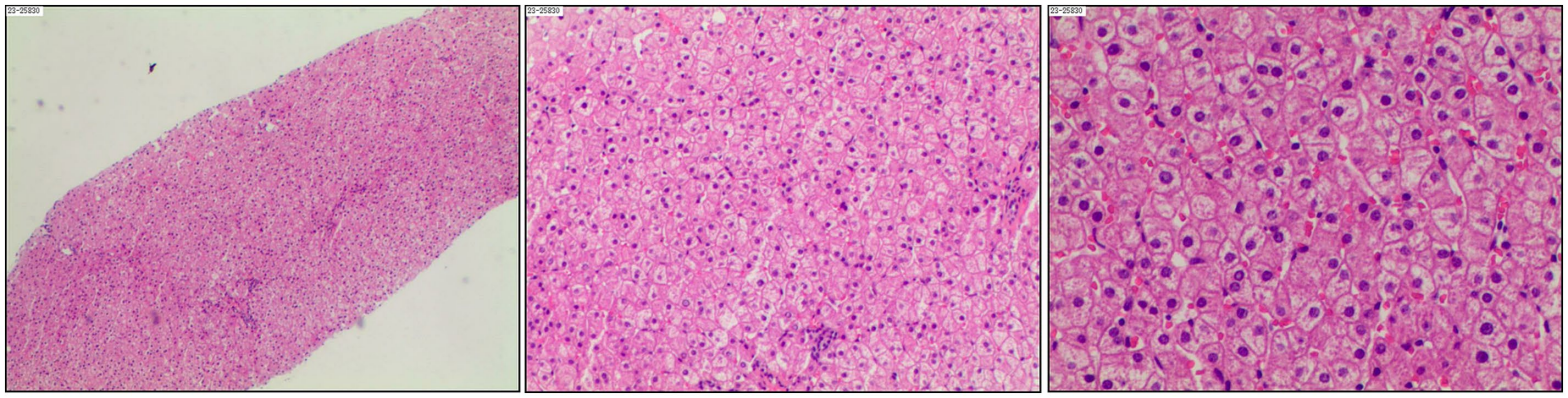
Liver biopsy of the patient in August 2023.

**Figure 2:**
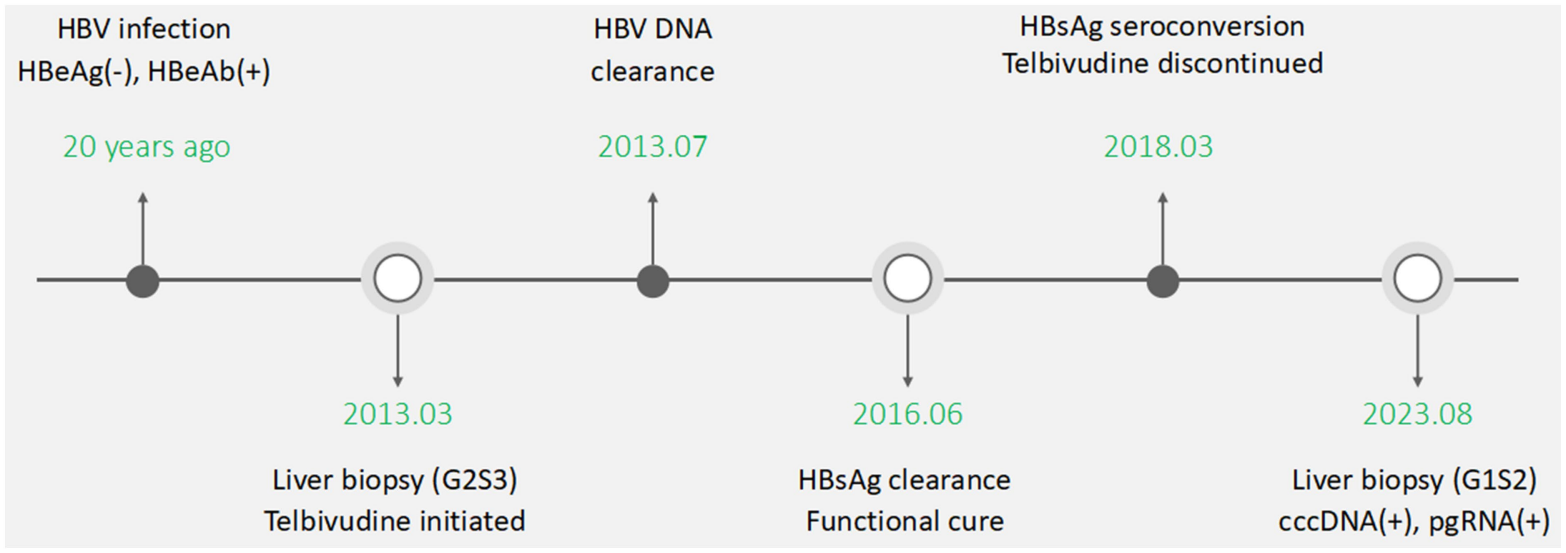
Treatment and follow-up management of the patient.

On 8 August 2023, the patient attended the Third Affiliated Hospital of Sun Yat-sen University for follow-up when she was 36 years old. Her blood HBV pgRNA level was “<25 copies/mL” (SUPBIO Biotechnology, Guangzhou, China), and liver stiffness by transient elastography test was 6.0 kPa (normal range: <7.0 kPa). The AFP was negative. Abdomen ultrasonic examination showed scattered patchy abnormal hyper-echoic masses in the liver and normal spleen. On 24 August 2023, a liver biopsy targeting the masses was performed again to assess the properties of hepatic occupying lesions and identify the existence of biomarkers of HBV in the hepatic cells, such as HBsAg, hepatitis B core antigen (HBcAg), cccDNA, and pgRNA. The structure of the hepatic lobule was preserved. Some hepatocytes showed mild hydropic degeneration. Spotty necrosis was observed scattered throughout the hepatic lobule. Cholestasis could not be observed within the hepatocytes and bile canaliculi. Some portal tracts were slightly enlarged, with mild to moderate lymphocyte infiltration. Piecemeal necrosis and bridging necrosis were not seen. Focal fibrosis could be found, and there was no obvious bile ductule reaction. The results showed chronic hepatitis with inflammation staging “G1” and fibrosis staging “S2” (Figure 1). Negative HBsAg and HBcAg and positive HBV cccDNA (2.70 copies/cell) and pgRNA (130.40 copies/mL) were found in hepatic cells (SUPBIO Biotechnology, Guangzhou, China). A sterilizing cure of CHB was not achieved, despite a functional cure having been achieved for over 7 years. On 24 January 2024, blood examinations showed normal ALT levels and negative HBsAg, HBV DNA, and HBV pgRNA. Neither Peg-IFN-*α* treatment nor NAs re-treatment was initiated. The treatment and follow-up management of the patient are shown in Figure 2. The laboratory results are shown in Table 1.

**Table 1:** Laboratory test results of the patient from treatment initiation to long-term follow-up.

Characteristics	March 2013 (telbivudine initiated)	July 2013 (HBV DNA clearance)	June 2016 (HBsAg clearance)	March 2018 (telbivudine discontinued)	August 2023	January 2024
Blood						
WBC, ×10^9^/L	7.80	8.12	7.53	9.04	8.58	
RBC, ×10^12^/L	5.70	5.14	5.84	6.38	6.02	
Hemoglobin, g/L	124	136	125	130	124	
Platelet, ×10^9^/L	152	137	139	192	241	
AST, U/L	24	26	29	22	17	19
ALT, U/L	20	16	19	13	11	10
TBIL, μmol/L	10.6	10.4	12	5.2	9.6	5.4
Albumin, g/L	47.0	47.4	46.6	47.7	47.8	47.8
AFP, ng/mL	Negative		1.65	0.905	1.34	1.24
HBsAg, IU/mL	Positive		<0.05	<0.05	<0.05	<0.05
HBsAb, mIU/mL	Negative		<2	76.04	29.097	
HBeAg	Negative				Negative	Negative
HBeAb	Positive				Positive	Negative
HBcAb	Positive				Positive	
HBV DNA, IU/mL	37,900	<100	<100	Not detected	Not detected	Not detected
pgRNA, copies/mL					<25	Not detected
Ultrasound						
Liver	Scattered patchy abnormal hyper-echoic masses	Scattered patchy abnormal hyper-echoic masses	Scattered patchy abnormal hyper-echoic masses	Scattered patchy abnormal hyper-echoic masses	Scattered patchy abnormal hyper-echoic masses	Scattered patchy abnormal hyper-echoic masses
Spleen	Slight enlargement	Slight enlargement	Slight enlargement	Slight enlargement	Normal	Normal
Liver biopsy						
Inflammation staging	G2				G1	
Fibrosis staging	S3				S2	
HBsAg	Positive				Negative	
HBcAg	Positive				Negative	
cccDNA, copies/cell					2.70	
pgRNA, copies/mL					130.40	

WBC: white blood cells; RBC: red blood cells; AST: aspartate aminotransferase; ALT: alanine aminotransferase; TBIL: total bilirubin; AFP: alpha-fetoprotein; HBsAg: hepatitis B surface antigen; HBsAb: hepatitis B surface antibody; HBeAg: hepatitis B e antigen; HBeAb: hepatitis B e antibody; HBcAb: hepatitis B core antibody; HBV: hepatitis B virus; DNA: deoxyribonucleic acid; pgRNA: pregenomic ribonucleic acid; HBcAg: hepatitis B core antigen; cccDNA: covalently closed circular DNA.

## Discussion

In this case, HBV-related biomarkers, including HBsAg, HBV DNA, and HBV pgRNA in the blood sample and HBsAg and HBcAg in the liver tissue, were negative. However, HBV cccDNA and pgRNA in the liver tissue remained positive, despite a functional cure being achieved for over 7 years (from June 2016 to January 2024) and HBsAg seroconversion achieved for over 5 years (from March 2018 to January 2024). It indicates that HBsAg seroconversion only reflects the silence but not the elimination of cccDNA in CHB patients. Since sterilizing the cure of CHB is hard to achieve and there are currently no new biomarkers reflecting the elimination of cccDNA, liver biopsy and tissue cccDNA examination are essential for confirmation of it.

The persistence of HBV cccDNA within the hepatocytes is a well-known problem for reservoirs of HBV, and current knowledge on cccDNA formation and degradation is still very limited (11). Traces of HBV are often detectable in patients for years after recovery from acute hepatitis, under the control of cytotoxic T-lymphocyte response (12, 13). Low but persistent levels of cccDNA and pgRNA were found in hepatocytes in CHB patients achieving a functional cure (14). HBV can maintain its occupancy of host cells in CHB patients in the form of episomal cccDNA or as integrated DNA while avoiding excessive antigen-specific immune responses (15). To achieve the goal of a functional cure or sterilizing cure for HBV infection, combination therapies that target different stages of the HBV life cycle and immunomodulatory threatments, provide hope and may be the ideal strategy for CHB patients (16). Novel therapies for the viral life cycle mainly include targeting virus entry, capsid assembly, polymerase, cccDNA, HBsAg, and viral transcript, while novel therapies for the host immune mainly include toll-like receptor agonists, checkpoint inhibitors, therapeutic vaccines, genetically engineered T-cells, stimulation of B-cell immune response, and metabolic modulation (16, 17). A first-in-class orally available cccDNA inhibitor has been identified recently to substantially reduce the level of cccDNA in mice models (18). It encourages us to explore new approaches for curing CHB.

The therapeutic management and long-term prognosis remain unclear for CHB patients with HCC family history and functional cure after antiviral treatment. There are no recommendations for this type of patient from the guidelines (6–9). In this case, inflammation and fibrosis staging by liver biopsy improved after NA discontinuation. AFP levels remained negative, and there was no occurrence of liver cirrhosis or HCC during the 5-year follow-up. NA treatment can be discontinued after a functional cure is achieved and maintained in CHB patients. However, long-term NA treatment is recommended for CHB patients with HCC family history (6). NA treatment is reported to reduce the incidence rate and recurrence rate of HCC (19, 20). Whether the patient in this case should be treated with long-term NA treatment requires further consideration by clinical physicians, but surveillance of HCC occurrence in long-term follow-up is necessary. A cohort study for surveillance of CHB patients with a family history of HCC and functional cure is needed, in order to confirm their long-term prognosis.

In conclusion, new approaches for curing CHB may be achieved in the future. Since there are currently no new biomarkers reflecting the elimination of cccDNA, active surveillance of HBV cccDNA and pgRNA in liver tissue is important for sterilizing the cure of CHB and the risk of HCC, although a functional cure is achieved.

## Data Availability

The raw data supporting the conclusions of this article will be made available by the authors, without undue reservation.
